# Hand hygiene knowledge, beliefs, and practices among healthcare professionals in the primary healthcare centers in Riyadh, Saudi Arabia: a cross-sectional study

**DOI:** 10.25122/jml-2025-0007

**Published:** 2025-04

**Authors:** Shuaa Rahail Alanazi, Abeer Jassam Alanazi, Mona Salem Alanazi, Fatima Rashid Alenazi, Nessreen Mohammed Algushiry, Ebtisam Awadh Alotaibi, Amerah Ayedh Alenazi, Hind Farhan Alanazi, Mariyyaha Mahdi Alanazi, Hani Rahail Alanazi, Mshari Anwar Alanezi, Latifah Rahail Alanazi

**Affiliations:** 1 AlRawda Primary Healthcare Center-2, Riyadh Second Health Cluster, Ministry of Health, Riyadh, Saudi Arabia; 2 Dental Center East Riyadh, Riyadh Second Health Cluster, Ministry of Health, Riyadh, Saudi Arabia; 3 Narjis Primary Healthcare Center, Riyadh Second Health Cluster, Ministry of Health, Riyadh, Saudi Arabia; 4 Almanar Primary Healthcare Center, Riyadh Second Health Cluster, Ministry of Health, Riyadh, Saudi Arabia

**Keywords:** Beliefs, hand hygiene, healthcare professionals, knowledge, practices, primary healthcare centers

## Abstract

Hand hygiene (HH) is vital for preventing healthcare-associated infections and ensuring patient safety. This study evaluated the knowledge, beliefs, and practices of healthcare professionals in primary healthcare centers (PHCs) in Riyadh, Saudi Arabia. A cross-sectional descriptive study was conducted using an online validated questionnaire based on the World Health Organization Hand Hygiene Knowledge Questionnaire. Data were analyzed to identify gaps and patterns across professional roles and demographics. A total of 221 healthcare professionals participated, 76% women and 24% men, 57% aged 30–39 years. Most had over 15 years of experience (33%), with nurses comprising the largest professional group. Nearly all participants (91%) had received HH training in the past 3 years, and 88% reported routine use of alcohol-based hand sanitizer. However, only 67% correctly identified the minimum time for alcohol-based sanitizer to kill germs, and misconceptions about hand rubbing efficacy persisted. The primary route of germ transmission was identified as healthcare workers' unclean hands. Most participants agreed that hand rubbing is faster than handwashing and emphasized HH after patient contact, exposure to body fluids, or contact with the patient’s surroundings. Nearly all endorsed using both hand rubbing and handwashing for specific scenarios, such as before injections. This study highlights strong HH knowledge and adherence among healthcare workers in Riyadh PHCs, reflecting effective training programs. However, persistent misconceptions and knowledge gaps regarding germ transmission and hand rubbing efficacy require targeted interventions.

## INTRODUCTION

Hand hygiene (HH) is a fundamental practice in infection prevention and control, with its importance emphasized by the World Health Organization (WHO) as a key measure to reduce healthcare-associated infections [1]. For healthcare professionals, particularly in primary healthcare centers (PHCs), effective HH is essential to protect patients and prevent the transmission of infectious diseases within healthcare settings. Despite global guidelines and widespread education campaigns, HH compliance among healthcare workers remains challenging in many countries, including Saudi Arabia. Suboptimal HH knowledge and practices are key barriers to effective infection control [2,3].

In Saudi Arabia, various studies have examined HH awareness among healthcare workers, revealing that while most healthcare professionals understand the importance of HH, gaps in knowledge and non-compliance with recommended practices are prevalent. In Riyadh, the capital of Saudi Arabia, these issues have been particularly notable in the PHCs, where high patient volumes and close interactions increase the risk of infection transmission. While healthcare workers were generally aware of the critical moments for HH, compliance rates were lower than expected, especially during routine patient care [4].

Recent research has also highlighted the need for enhanced education and targeted interventions to bridge these knowledge gaps. Although healthcare workers were knowledgeable about HH guidelines, significant barriers to proper implementation existed, such as lack of time, heavy workload, and inadequate access to necessary supplies [5]. Continuous training and institutional support to improve HH practices, especially in high-risk healthcare environments like primary care, is vital [6].

Despite the growing body of research, there is still limited focus on the specific knowledge and practices of healthcare professionals in PHCs. Understanding HH behavior in this setting is crucial for designing effective interventions that promote consistent compliance. Therefore, this study aimed to assess the level of HH knowledge, beliefs, and practices among healthcare professionals in the PHCs located in Riyadh using the WHO hand hygiene knowledge questionnaire for healthcare workers [7].

## MATERIAL AND METHODS

### Study design and setting

This cross-sectional descriptive study assessed the HH knowledge, beliefs, and practices of healthcare workers from PHCs in Riyadh, Saudi Arabia. The selected primary healthcare centers were in various districts of Riyadh, representing both urban and suburban areas, to ensure a diverse sample of healthcare workers.

### Study participants

The target population for this study was all healthcare professionals working in PHCs in Riyadh. Eligible participants were healthcare workers involved in direct patient care, including physicians, nurses, pharmacists, dentists, and other allied health professionals, such as laboratory specialists or radiologists. Participants were selected using a convenience sampling method, with a target sample size of 250 healthcare workers, calculated to provide adequate statistical power (5% margin of error and 90% confidence interval) in HH knowledge across various demographic groups. The inclusion criteria comprised all healthcare professionals actively working in PHCs who were willing to participate in the study and had at least six months of experience in patient care. Exclusion criteria involved healthcare workers without direct patient contact (e.g., administrative staff).

### Data collection instrument

The data were collected using the WHO HH knowledge questionnaire for healthcare workers, a validated tool widely used to assess the HH knowledge of healthcare workers [7]. This questionnaire was adapted for the local context and used in various studies worldwide to assess theoretical knowledge and practical understanding of HH protocols. In addition to the demographic data (gender, age range, years of experience, and specialty), the WHO questionnaire consists of multiple-choice questions divided into sections addressing:

1. **Basic HH knowledge:** This section evaluates participants' understanding of the importance of HH and the basic principles involved in proper handwashing.
2. **Critical moments for HH:** This section examines knowledge of key moments when HH is essential, such as before patient contact, after body fluid exposure, and after touching surfaces.
3. **HH techniques:** This section assesses participants’ knowledge about the correct techniques for hand hygiene, including the duration and method of handwashing and the use of alcohol-based hand rubs.
4. **Infection control guidelines:** This section focuses on knowledge regarding institutional HH policies, national guidelines, and common barriers to compliance.

Fourteen questions from the WHO questionnaire were selected and translated into Arabic following a discussion among experts to ensure they aligned with the study's objectives and were clear and understandable for all participants (supplementary file). A pilot study was conducted with 15 healthcare workers to assess the clarity and validity of the Arabic version, and any necessary adjustments were made before the full-scale study. The reliability of the Arabic questionnaire was assessed using statistical methods, including Cronbach’s alpha and inter-rater reliability. A Cronbach’s α value of 0.80 was obtained, indicating good internal consistency. No modifications were made to the Arabic version of the questionnaire.

Supplementary Material

### Data collection and statistical analysis

The questionnaires were prepared electronically using Google Forms and distributed to the target participants via email and WhatsApp. The first page of the questionnaire was designed to explain the study to all potential participants and obtain their informed consent. Participants were assured of confidentiality and anonymity in the study. Each participant was asked to complete the questionnaire independently. Data were entered and analyzed using SPSS version 25. Descriptive statistics, including frequencies and percentages, were used to summarize the participants' responses to the questionnaire.

## RESULTS

A total of 221 participants were included in the study, comprising 53 men (24%) and 168 women (76%). Of the total, 125 (57%) participants were aged 30–39 years, 55 (25%) were aged 40–49 years, and 31 (14%) were aged 20–29 years. Only 10 (4.5%) participants were aged 50 years and older (Table 1). Most participants (*n* = 73, 33%) had an experience of more than 15 years, followed by 10–15 years of experience (*n* = 69, 31%). Nurses represented more than half of the participants (Table 1). Most participants (*n* = 201, 91%) had training on HH in the last 3 years. The routine use of alcohol-based hand sanitizer was reported by 195 (88%) participants. When asked about the minimum amount of time required for an alcohol-based hand rub to kill most germs effectively, 148 participants (67%) selected 20 seconds, while 46 (20.8%) believed 10 seconds was sufficient (Table 2).

**Table 1:** Demographic characteristics of participants

Characteristic	Frequency (n)	Percentage (%)
**Gender**		
Men	53	24.0
Women	168	76.0
**Age group**		
20–29 years	31	14.0
30–39 years	125	57.0
40–49 years	55	25.0
≥50 years	10	4.5
**Years of experience**		
> 15 years	73	33.0
10–15 years	69	31.0
6–9 years	23	10.0
4–6 years	19	9.0
1–3 years	37	17.0
**Specialty**		
Nursing	114	51.4
Medicine	32	14.4
Allied health professions	32	14.4
Dentistry	29	13.1
Pharmacy	14	6.3

**Table 2:** Training and hand hygiene practices

Aspect	Frequency (*n*)	Percentage (%)
**Training on hand hygiene**		
Yes	201	91.0
No	20	9.0
**Routine use of alcohol sanitizer**		
Yes	195	88.0
No	26	12.0
**Response to perceived minimum time for effective hand sanitization**		
20 seconds (True)	148	67.0
10 seconds	46	20.8
3 seconds	10	4.5
1 minute	17	7.7

A total of 146 participants (66.1%) identified the unclean hands of healthcare workers as the main route of cross-transmission of potentially harmful germs between patients in healthcare facilities. This was followed by air circulation within the healthcare facility (*n* = 55, 24.9%) and sharing non-invasive objects (i.e., stethoscopes, pressure cuffs, etc.) between patients (*n* = 14, 6.3%). Only six participants (2.75%) believed patients’ exposure to colonized surfaces (i.e., beds, chairs, tables, floors) is the main route of cross-transmission of potentially harmful germs between patients in a healthcare facility (Table 3). The environments (surfaces) of the healthcare facility were selected as the most frequent source of germs responsible for healthcare-associated infections by 111 participants (50.2%). Of the total, 73 (33%) believed air in the healthcare facility is the most frequent source of germs responsible for healthcare-associated infections, followed by the water system in the healthcare facility (*n* = 21, 9.5%) and germs already present on or within the patient (*n* = 16, 7.2%) (Table 3).

**Table 3:** Perceived main routes of cross-transmission of germs in healthcare facilities and most frequent source of germs responsible for healthcare-associated infections

Route of cross-transmission	Frequency (*n*)	Percentage (%)
Healthcare workers' hands when not clean	146	66.1
Air circulating in the healthcare facility	55	24.9
Sharing non-invasive objects (e.g., stethoscopes, pressure cuffs)	14	6.3
Patients’ exposure to colonized surfaces	6	2.75
**Source of germs**		
Healthcare facility environment (surfaces)	111	50.2
Air in the healthcare facility	73	33.0
Water system in the healthcare facility	21	9.5
Germs already present on or within the patient	16	7.2

Most participants (*n* = 151, 68.3%) believed hand rubbing is more rapid for hand cleansing than handwashing. More than half of the participants (*n* = 136, 61.5%) agreed that hand rubbing causes skin dryness more than handwashing. The statement “hand rubbing is more effective against germs than handwashing” was considered false by 131 participants (59.3%). Of the total, 180 (81.4%) participants agreed that handwashing and hand rubbing are recommended to be performed in sequence (Table 4). When asked about specific hand hygiene actions that prevent the transmission of germs to healthcare workers, 208 (94.1%) agreed after touching a patient, 206 (92.3%) immediately after the risk of body fluid exposure, 195 (88.2%) immediately before a clean/aseptic procedure, and 204 (92.3%) after exposure to the immediate surroundings of a patient (Table 4).

**Table 4:** Participants' beliefs and practices regarding hand hygiene

Statement or Action	Frequency (*n*)	Percentage (%)
**Beliefs about hand rubbing vs. handwashing**		
Hand rubbing is more rapid for hand cleansing than handwashing	151	68.3
Hand rubbing causes skin dryness more than handwashing	136	61.5
Hand rubbing is more effective against germs than handwashing (False)	131	59.3
Handwashing and hand rubbing are recommended to be performed in sequence	180	81.4
**Hand hygiene actions to prevent germ transmission to healthcare workers**		
After touching a patient	208	94.1
Immediately after a risk of body fluid exposure	206	92.3
Immediately before a clean/aseptic procedure	195	88.2
After exposure to the immediate surroundings of a patient	204	92.3

Again, the majority of participants (*n* = 210, 95%) believed that both hand rubbing and handwashing are required before palpating the abdomen and before administering an injection. Similarly, 217 (98%) indicated that both methods are necessary after emptying a bedpan, removing examination gloves, and making a patient's bed. Most participants (*n* = 219, 99%) agreed that rubbing and washing hands is necessary after visible exposure to blood. Most participants (*n* = 187, 85%) agreed that wearing jewelry should be avoided due to its association with an increased likelihood of hand colonization by harmful germs. Additionally, artificial fingernails (*n* = 198, 90%), damaged skin (*n* = 181, 82%), and regular use of hand cream (*n* = 113, 51%) were identified by participants as factors associated with a higher risk of microbial colonization (Table 5).

**Table 5:** Participants' beliefs and practices on hand hygiene and factors increasing germ colonization

Statement or Action	Frequency (*n*)	Percentage (%)
**Hand hygiene method beliefs**		
Rubbing and washing hands before palpation of the abdomen and giving an injection	210	95.0
Rubbing and washing hands after emptying a bedpan, removing gloves, and making bed	217	98.0
Rubbing and washing hands after visible exposure to blood	219	99.0
**Factors to avoid due to increased germ colonization**		
Wearing jewelry	187	85.0
Artificial fingernails	198	90.0
Damaged skin	181	82.0
Regular use of hand cream	113	51.0

## DISCUSSION

This study assessed healthcare workers’ knowledge, beliefs, and practices regarding HH in PHCs in Riyadh, Saudi Arabia. This study provided insights into adherence to international HH guidelines and identifying areas for improvement.

### Demographics and training

Most participants were women (76%) aged 30–39 (57%). Similar demographic trends have been reported, as nursing and related professions often have a predominantly female workforce [8]. Most participants (91%) received formal HH training within the last 3 years, aligning with the WHO guidelines emphasizing regular training for effective HH compliance [9]. This finding is consistent with a report on HH programs in Saudi Arabia, where 91% of healthcare workers had received HH training within 2 years, reflecting the importance of continuous education in maintaining awareness [10].

### Knowledge of HH practices

Approximately 67% of participants correctly identified the minimum time (20 seconds) required for effective hand rubbing, aligning with the WHO recommendations [9]. However, 20.8% underestimated this time, indicating potential gaps in their awareness. Similar results were observed in a study in Jeddah, Saudi Arabia, where 30% of participants were unaware of the recommended duration for handrubbing [11]. Internationally, gaps in knowledge about HH duration have also been documented, emphasizing the universal need for reinforcing basic concepts through regular training [12,13].

### Perceived sources of germ transmission

Most participants (66.1%) identified unclean healthcare professionals’ hands as the main route of germ cross-transmission, which aligns with evidence that hands are the primary vectors for healthcare-associated infections [14,15]. However, misconceptions persisted, with 24.9% attributing the primary source of healthcare-associated infections to air circulation. Similar misunderstandings were observed, where healthcare workers attributed healthcare-associated infections to environmental factors over direct transmission mechanisms [16].

### Compliance and adherence

Compliance rates in this study were high, with 94.1% performing HH after patient contact and 92.3% after exposure to patient surroundings. These rates surpass international averages, where adherence to specific HH moments often falls below 70% [17]. The high compliance reported here reflects the impact of infection control policies in Saudi Arabia, including the national HH campaign, which has significantly improved HH practices [18,19].

### Beliefs about hand rubbing versus handwashing

While most participants (68.3%) believed hand rubbing was quicker than handwashing, over half (59.3%) did not believe it was more effective against germs. This reflects persistent confusion about the complementary roles of these methods. However, 81.4% agreed that handwashing and rubbing should be performed sequentially, reflecting adherence to WHO guidelines [9]. Similar misconceptions were recognized about the importance of using both techniques sequentially.

### Factors increasing germ colonization

Participants demonstrated strong awareness of factors increasing hand colonization, with 90% identifying artificial fingernails, 85% identifying jewelry, and 82% identifying damaged skin. These findings are consistent with global recommendations highlighting the risks of such practices [9,20]. However, only 51% associated regular hand cream use with colonization risk, indicating a gap in understanding product interactions with antiseptics. This pattern was similarly observed in Saudi healthcare facilities [10]. In conclusion, several studies in Saudi Arabia report high HH training rates but identify gaps in sustained practice adherence and knowledge among less experienced healthcare workers [18]. Internationally, HH compliance is influenced by workload, institutional policies, and cultural attitudes, with adherence rates varying widely between regions [21]. The high compliance and training rates observed in this study reflect the success of Saudi infection control efforts, particularly in larger healthcare facilities.

### Study limitations

This study has a few limitations. To begin with, the sample size was relatively small, and participants in the PHCs may not represent the broader population of healthcare workers in healthcare facilities in other Saudi regions. Therefore, the results of this study might not be generalizable to other healthcare settings, such as tertiary hospitals. In addition, data on practices, such as HH compliance, often rely on self-reported questionnaires and are prone to overestimation or social desirability bias. Participants may report what they perceive as expected rather than their actual practices. Finally, the study only provides insights into the current state of knowledge or practices but does not account for changes over time, such as variations in compliance during different shifts or after interventions.

## CONCLUSION

This study highlights strong HH knowledge and adherence among healthcare workers in the PHCs in Riyadh, reflecting the effectiveness of training programs. However, persistent misconceptions about the efficacy of hand rubbing and knowledge gaps regarding factors influencing colonization require targeted interventions. Future research should explore regional differences in HH compliance and the impact of institutional policies on healthcare workers’ practices.

## Data Availability

Further data is available from the corresponding author upon reasonable request.

## References

[1] World Health Organization (2009). WHO guidelines on hand hygiene in health care. https://www.who.int/publications/i/item/9789241597906.

[2] Kim J, Yu SN, Jeong YS, Kim JH, Jeon MH, Kim T (2023). Hand hygiene knowledge, attitude, barriers and improvement measures among healthcare workers in the Republic of Korea: A cross-sectional survey exploring interprofessional differences. Antimicrob Resist Infect Control.

[3] Mohaithef M (2020). Assessing hand hygiene practices among nurses in the Kingdom of Saudi Arabia. Open Public Health J.

[4] Alanazi KSN, Alanazi FH, Alanazi YL, Alanazi KS, Alanazi IH, Alatawi RA (2024). Knowledge, attitude, and practice of hand hygiene among health workers in primary healthcare centers in Saudi Arabia: A systematic review. Int J Med Dev Ctries.

[5] Alshagrawi S, Alhodaithy N (2024). Determinants of hand hygiene compliance among healthcare workers in intensive care units: A qualitative study. BMC Public Health.

[6] Mahfouz AA, Abolyazid A, Al-Musa HM, Awadallah NJ, Faraheen A, Khalil S (2017). Hand hygiene knowledge of primary health care workers in Abha city, South Western Saudi Arabia. J Family Med Prim Care.

[7] World Health Organization (2009). WHO hand hygiene knowledge questionnaire for healthcare workers. https://cdn.who.int/media/docs/default-source/integrated-health-services-(ihs)/hand-hygiene/monitoring/surveyform/hand-hygiene-knowledge-questionnaire.doc?sfvrsn=dbb4da65_2.

[8] Saudi Health Council (2019). The nursing workforce in Saudi Arabia: Challenges and opportunities. https://shc.gov.sa/Arabic/Documents/KSA_Nursing_Challenges_and_Opportunties_pub_6-22-20.pdf.

[9] World Health Organization (2018). My five moments for hand hygiene. https://www.who.int/publications/m/item/five-moments-for-hand-hygiene.

[10] Mutashish S, Al Bujayr A, Al Saihati I, Al Matraf N (2020). General directorate of infection prevention control: Hand hygiene program. https://jed-s3.bluvalt.com/psj1-ifn-s3-ifn01/files/01/Hand-Hygiene/Hand-Hygiene-Annual-Report-2020.pdf.

[11] Bakarman MA, Baig M, Malik AA, Gazzaz ZJ, Mostafa MM, Zayes MA (2019). Hand hygiene knowledge and attitude of medical students in western Saudi Arabia. PeerJ.

[12] Pittet D (2001). Improving adherence to hand hygiene practice: A multidisciplinary approach. Emerg Infect Dis.

[13] Erasmus V, Daha TJ, Brug H, Richardus JH, Behrendt MD, Vos MC (2010). Systematic review of studies on compliance with hand hygiene guidelines in hospital care. Infect Control Hosp Epidemiol.

[14] Allegranzi B, Pittet D (2009). Role of hand hygiene in healthcare-associated infection prevention. J Hosp Infect.

[15] Kampf G, Löffler H, Gastmeier P (2009). Hand hygiene for the prevention of nosocomial infections. Dtsch Arztebl Int.

[16] Ministry of Health (2023). Healthcare-associated outbreak management manual. https://www.moh.gov.sa/Ministry/Rules/Documents/Healthcare-Associated-Outbreak-Management-Manual.pdf.

[17] World Health Organization (2025). Hand hygiene: The evidence for clean hands. https://www.who.int/teams/integrated-health-services/infection-prevention-control/hand-hygiene#:~:text=Available%20evidence%20shows%20that%20compliance,(64.5%25%20vs%209.1%25).

[18] Ministry of Health (2022). National hand hygiene campaign. https://www.moh.gov.sa/en/HealthAwareness/EducationalContent/PublicHealth/Pages/Handwashing.aspx.

[19] Al-Tawfiq JA, Abed MS, Al-Yami N, Birrer RB (2013). Promoting and sustaining a hospital-wide, multifaceted hand hygiene program resulted in significant reduction in health care-associated infections. Am J Infect Control.

[20] Boyce J, Pittet D, Healthcare Infection Control Practices Advisory Committee; HICPAC/SHEA/APIC/IDSA Hand Hygiene Task Force (2002). Guideline for hand hygiene in healthcare settings. MMWR Recomm Rep.

[21] Sax H, Allegranzi B, Chraïti MN, Boyce J, Larson E, Pittet D (2009). The World Health Organization hand hygiene observation method. Am J Infect Control.

