# Supplementary material for: Hand hygiene knowledge, beliefs, and practices among healthcare professionals in the primary healthcare centers in Riyadh, Saudi Arabia: a cross-sectional study
Source: J Med Life. 2025 Apr;18(4):332–7. doi: 10.25122/jml-2025-0007 (PMC12094307; doi:10.25122/jml-2025-0007)
Supplement: Supplementary file 1 [file JMedLife-18-332-s001.pdf]

A. DEMOGRAPHIC INFORMATION:

1. Gender

- ☐ Male
- ☐ Female

2. Age

- ☐ 20–29
- ☐ 30–39
- ☐ 40–49
- ☐ 50–59
- ☐ ≥60

3. Years of experience

- ☐ 1–3
- ☐ 4–6
- ☐ 7–9
- ☐ 10–15
- ☐ ≥16

4. Specialty

- ☐ Medicine
- ☐ Dentistry
- ☐ Nursing
- ☐ Pharmacy
- ☐ Allied Health professional

B. BASIC HH KNOWLEDGE

5. Did you receive formal training in hand hygiene in the last three years?

- ☐ Yes
- ☐ No

6. Do you routinely use an alcohol-based handrub for hand hygiene?

- ☐ Yes
- ☐ No

7. Which of the following is the main route of cross-transmission of potentially harmful germs between patients in a healthcare facility? (*tick one answer only*)

- ☐ Health-care workers' hands when not clean
- ☐ Air circulating in the hospital
- ☐ Patients' exposure to colonized surfaces (i.e., beds, chairs, tables, floors)
- ☐ Sharing non-invasive objects (i.e., stethoscopes, pressure cuffs, etc.) between patients

8. What is the most frequent source of germs responsible for healthcare-associated infections? (*tick one answer only*)

- ☐ The hospital's water system
- ☐ The hospital air
- ☐ Germs already present on or within the patient
- ☐ The hospital environment (surfaces)

© 2025 by the authors. This article is an open access article distributed under the terms and conditions of the Creative Commons Attribution (CC BY 4.0) license.

C. CRITICAL MOMENTS FOR HH:

9. Which of the following hand hygiene actions prevents transmission of germs *to the patient*?

9.a. Before touching a patient

- ☐ Yes
- ☐ No

9.b. Immediately after a risk of body fluid exposure

- ☐ Yes
- ☐ No

9.c. After exposure to the immediate surroundings of a patient

- ☐ Yes
- ☐ No

9.d. Immediately before a clean/aseptic procedure

- ☐ Yes
- ☐ No

10. Which of the following hand hygiene actions prevents transmission of germs *to the healthcare worker*?

10.a. After touching a patient

- ☐ Yes
- ☐ No

10.b. Immediately after a risk of body fluid exposure

- ☐ Yes
- ☐ No

10.c. Immediately before a clean/aseptic procedure

- ☐ Yes
- ☐ No

10.d. After exposure to the immediate surroundings of a patient

- ☐ Yes
- ☐ No

D. HAND HYGIENE TECHNIQUES

11. What is the minimal time needed for alcohol-based handrub to kill most germs on your hands? (*tick one answer only*)

- ☐ 20 seconds
- ☐ 3 seconds
- ☐ 1 minute
- ☐ 10 seconds

© 2025 by the authors. This article is an open access article distributed under the terms and conditions of the Creative Commons Attribution (CC BY 4.0) license.

12. Which of the following statements on alcohol-based handrub and handwashing with soap and water are true?

12.a. Handrubbing is more rapid for hand cleansing than handwashing

- ☐ True
- ☐ False

12.b. Handrubbing causes skin dryness more than handwashing

- ☐ True
- ☐ False

12.c. Handrubbing is more effective against germs than handwashing

- ☐ True
- ☐ False

12.d. Handwashing and handrubbing are recommended to be performed in sequence

- ☐ True
- ☐ False

E. INFECTION CONTROL GUIDELINES

13. Which type of hand hygiene method is required in the following situations?

13.a. Before palpation of the abdomen

- ☐ Rubbing
- ☐ Washing
- ☐ None

13.b. Before giving an injection

- ☐ Rubbing
- ☐ Washing
- ☐ None

13.c. After emptying a bedpan

- ☐ Rubbing
- ☐ Washing
- ☐ None

13.d. After removing examination gloves

- ☐ Rubbing
- ☐ Washing
- ☐ None

13.e. After making a patient's bed

- ☐ Rubbing
- ☐ Washing
- ☐ None

© 2025 by the authors. This article is an open access article distributed under the terms and conditions of the Creative Commons Attribution (CC BY 4.0) license.

13.h. After visible exposure to blood

- ☐ Rubbing
- ☐ Washing
- ☐ None

14. Which of the following should be avoided, as associated with the increased likelihood of colonization of hands with harmful germs?

14.a. Wearing jewellery

- ☐ True
- ☐ False

14.b. Damaged skin

- ☐ True
- ☐ False

14.c. Artificial fingernails

- ☐ True
- ☐ False

14.d. Regular use of a hand cream

- ☐ True
- ☐ False

© 2025 by the authors. This article is an open access article distributed under the terms and conditions of the Creative Commons Attribution (CC BY 4.0) license.
